# Employers most desirable attributes in early-career physiotherapists: a content analysis of job advertisements

**DOI:** 10.1186/s12913-024-11470-6

**Published:** 2024-09-06

**Authors:** R. McAleer, L. Hanson, A. Kenny

**Affiliations:** 1 La Trobe Rural Health School, La Trobe University, Bendigo, VIC Australia; 2 Violet Vines Marshman Centre for Rural Health Research, La Trobe Rural Health School, La Trobe University, Bendigo, VIC Australia; 3 University of Lincoln, Lincoln, UK

**Keywords:** Physiotherapy, New graduate, Early-career, Attributes, Employability skills, Rural, Recruitment, Retention

## Abstract

**Background:**

Ensuring a sufficient, appropriately qualified health workforce is of global concern. Understanding the attributes that employers seek is critical in recruitment, retention, and educational design. In physiotherapy, there is a dearth of evidence on desirable attributes that employers seek from early-career physiotherapists. This study directly addresses this gap. The aims of this study were to identify the characteristics of the jobs advertised for early-career physiotherapists in Australia; determine which attributes were most desired when employing an early-career physiotherapist; and identify if there were differences in the attributes required based upon workplace location.

**Methods:**

New graduate and early-career physiotherapy job advertisements were collected for six months from 1st October 2020 until 31st March 2021 from SEEK.com.au, a large online employment marketplace that operates across ten countries in the Asia Pacific and Latin America. Job advertisements needed to specify new graduate or early-career physiotherapist eligibility and be located within Australia. Data extraction were completed using QuestionPro^®^. The Modified Monash Model was used to classify rurality of job location. Job advertisements were analysed descriptively and using content analysis to identify attributes.

**Results:**

The search yielded 578 job advertisements with the greatest number collected in October 2020 (25.3%). Of the advertisements, 428 (74.0%) were in metropolitan locations (Modified Monash 1), 47 (8.1%) were in regional (Modified Monash 2), 99 (17.1%) were in rural locations (Modified Monash 3–5), and 4 (0.8%) were in remote locations (Modified Monash 6–7). Most roles were in private practice (63.3%) or aged care (21.7%). The top five attributes requested by employers were client focus, communication and interpersonal skills, team player, willingness to learn, and being able to build rapport, relationships, and networks. Academic results, resilience, and empathy were the least requested attributes. Differences in requested employability attributes increased with rurality.

**Conclusions:**

This study addresses the current knowledge about attributes sought by employers for early-career physiotherapists. The most prevalent attributes requested were client focus and communication and interpersonal skills. This exploration of attributes can help to better prepare graduates for their first roles, align expectations, and increase understanding of priorities for entry level university programs, as well as identify priorities for support during transition to practice. Desired attributes should be clearly defined by employers in recruitment and retention processes.

**Supplementary Information:**

The online version contains supplementary material available at 10.1186/s12913-024-11470-6.

## Background

This study addressed a major gap in knowledge of the attributes that employers seek from early-career physiotherapists and differences in the attributes required based on workplace location. The recruitment and retention of appropriately qualified health professionals is essential for achieving universal health coverage [1, 2], improved health outcomes, and optimisation of an independent, productive population, that is living longer with a better quality of life [3]. Globally, universal health coverage and positive health outcomes are severely impacted by ongoing workforce shortages of the allied health workforce [4]. In physiotherapy, there are high attrition rates [5], and inadequate supply of professionals to meet demand [6]. 

### Employability attributes

Central to building a sustainable physiotherapy workforce, is ensuring that the attributes valued by employers align with the attributes of potential employees. This is particularly critical for newly qualified physiotherapists, as misalignment of attributes required by employers with attributes of new graduates can impact recruitment, transition to practice, and retention [7]. 

While employability attributes have been referred to in a variety of ways [8] there are common features. Rios and colleagues [5] refer to 21st century skills as a combination of cognitive, interpersonal, and intrapersonal skills. Some authors refer to generic skills, capabilities, or key competencies [9]. Other terminology includes soft skills [10], non-academic attributes [11], non-cognitive attributes [12], and non-technical skills [13]. In this study, we define attributes as personal and professional characteristics, traits, and skills that allow an early-career physiotherapist to complete their job role efficiently and effectively, and we focus on the first three years of practice.

### The changing physiotherapy workforce

The early-career physiotherapy workforce is changing to meet population need. Once predominantly public hospital-based, early-career physiotherapists are now increasingly entering private practice environments [14] and community settings such as primary care [15]. Diverse and new employment opportunities increase the need to understand the attributes that employers seek, as this knowledge has the potential to inform the content of entry level university programs and priorities for mentoring and support of newly qualified employees to further develop these attributes.

Capturing differences in attributes required by employers in different settings may help to better tailor recruitment practices and establish realistic expectations for new graduates, particularly in rural and remote contexts. The complexity of the rural environment, and major difficulties in the recruitment and retention of allied health professionals, severely impacts service delivery and accessibility of equitable access to healthcare. [7, 16, 17] In a systematic review that examined the experience of early-career allied health professionals [16], the diversity of reasons for applying for rural and remote positions was documented. While some applying for a rural or remote position might have grown up in rural and remote locations and/or have families in these locations, opportunities for high levels of autonomous work, a diversity of clinical experiences, and the challenges of a steep learning curve can entice new graduates to rural and remote practice [16]. While some, might be committed to rural and remote locations, others may have little experience, and may seek a position because of a perception that rural and remote positions are easier to obtain than those in metropolitan areas and are less competitive [17]. 

Challenges of rural practice for a newly qualified allied health professional include lack of clinical and manager support, high workloads, stress, long hours, feelings of being thrown in the deep end, limited resources, barriers to professional development, lack of professional and personal boundaries, and social isolation. [7, 16, 17] Onnis [7] reinforced the critical role that recruitment advertising has in establishing employer/employee expectations in rural and remote locations and that misalignment of these expectations can lead to a ‘severe and emotionally charged response to an unfulfilled obligation …’ (p.26) with resignation the likely outcome. A high level of transparency about the complexity of the rural and remote practice location, likely challenges, and the attributes required is critical to retention [7, 17]. 

### Previous studies on attributes in physiotherapy

Previous studies in physiotherapy are limited. Studies have focused on employers’ perceptions of key factors contributing to workplace readiness in novice physiotherapists [18], the role of pre-registration curricula in preparing physiotherapists, and attributes including the most important skills and qualities identified by employers when selecting applicants for entry-level physiotherapy positions [19]. In a Delphi study by Martin and colleagues [20], the attributes of entry-level rural and remote physiotherapists were identified by expert physiotherapists. A set of 19 attributes for rural practice were developed, with 100% agreement on practising flexibly, providing effective care across the lifespan, proactively managing one’s own health and resilience to meet rural practice challenges, understanding roles of team members, and ongoing skill development in rural physiotherapy practice. Many of these attributes were about adaptation and an understanding of the environment and the associated challenges. In contrast, our study focused on the most desirable employability attributes of an individual, encompassing personal and professional characteristics. Other authors focus on one attribute, including clinical decision-making [21], clinical reasoning [22], reflective practice [23], communication and interpersonal skills [24], and empathy [25]. The focus of other authors has been on the differences between the attributes of novice and experienced physiotherapists’ [21, 23, 26, 27] or postgraduate physiotherapist attributes [28]. 

### Physiotherapy in Australia

Physiotherapy is the fourth largest regulated health profession in Australia, comprising 4.6% of the regulated health workforce [29] incorporating around 40,000 registered physiotherapists [30]. Most physiotherapists work within large cities in New South Wales, Victoria, and Queensland, where there are 145 physiotherapists for every 100,000 people, decreasing to 89 per 100,000 regionally, and 46 per 100,000 in remote areas [29]. 

In Australia, the Modified Monash Model is used to measure geographical remoteness and town size from categories 1 to 7. Modified Monash (MM) category 1 is a major city and MM 7 is very remote [31]. The Modified Monash Model was developed by the Australian Government, Department of Health [31, 32] to better guide workforce distribution of health professionals in regional, rural, and remote areas (Table 1).

**Table 1:** Modified Monash Model category descriptions [31]

MM Category	Description
**MM 1: Metropolitan areas**	Major cities accounting for 70% of Australia’s population
**MM 2: Regional centres**	Areas that are in/within a 20 km drive of a town with over 50,000 residents
**MM 3: Large rural towns**	Areas that are in/within a 15 km drive of a town between 15,000 to 50,000 residents
**MM 4: Medium rural towns**	Areas that are in/within a 10 km drive of a town with between 5,000 to 15,000 residents
**MM 5: Small rural towns**	All remaining rural towns
**MM 6: Remote communities**	Remote mainland areas AND remote islands less than 5kms offshore AND islands that are MM 5 with a population of less than 1,000 without bridges to the mainland
**MM 7: Very remote communities**	Very remote areas and all other remote island areas more than 5kms offshore

To this point, no authors have explored the characteristics of jobs advertised for early-career physiotherapists in Australia, the most desirable attributes when employing an early-career physiotherapist, and differences in the attributes required in different workplace locations. In this study, we address this gap.

### The study

#### Aims

The aims of the study were to:

1. identify the characteristics of the jobs advertised for early-career physiotherapists in Australia.
2. determine which attributes were most desired when employing an early-career physiotherapist.
3. identify if there was a difference in the attributes required in the early-career physiotherapist based upon workplace location.

### Study Design

This study is a content analysis of job advertisements from the online employment platform SEEK.com.au, and was guided by the following research questions:

1. What are the characteristics of the jobs advertised for early-career physiotherapists in Australia?
2. What are the advertised attributes required of early-career physiotherapists?
3. What are the most desirable attributes?
4. Is there a difference in the attributes required in the early-career physiotherapist based on workplace location?

Job advertisements are a guide for employers and prospective employees. They provide an overview of role expectations and required responsibilities, skills, knowledge, behaviours, and attributes or characteristics deemed important for the right candidate [33]. They provide potential employees with direction on whether their skills and experience might align with employer expectations. Job advertisements can be used to gauge the expectations and demands of different roles and required attributes to successfully meet employer expectations. [34, 35] In a study by Onnis [7], recruitment advertising was examined to assess the alignment of advertising content with factors that attracted the current health workforce to rural and remote practice. As our aim was to identify attributes sought by employers, and differences in attributes depending on workplace location, analysis of job advertisements was identified as a method to explore the dynamic nature of the labour market [34] and the attributes sought for practice. Job advertisements provide easily accessible information for use in education, research, and policymaking [33]. 

### Data collection

Physiotherapy job advertisements on SEEK.com.au were collected for six months from 1st October 2020 until 31st March 2021. In Australia, this period is the peak time for organisations to be advertising new graduate positions, and for new graduates to be looking for employment. In Australia, the university academic year generally aligns with the calendar year. Advertisements were included if they met the following criteria: advertisements must specify new graduate or early-career physiotherapist eligibility, positions must be in Australia, and the position may be advertised by an employer or through a third-party recruitment agency. Advertisements were excluded if the position excluded new graduate or early-career physiotherapists, the role was not specific to physiotherapists, such as health promotion roles, and those that advertised positions in countries other than Australia.

Two separate searches were completed by one author (RM). The search terms used for relevant job advertisements on SEEK.com.au were ‘new graduate physiotherapist’ and ‘early-career physiotherapist’ anywhere within Australia. Job alerts were placed on each search and the researcher received a daily email with the search yield. Each advertisement was scanned for the terminology ‘new graduate’ or ‘early-career physiotherapist’ and if present, was saved in Word format with the advertisement date. Advertisements were then saved based on location, that is, the state or territory of Australia.

### Data extraction

The QuestionPro^®^ [36] online survey software was used to extract data. If an advertisement was repeatedly advertised, data were only extracted once. A customised 103 question data extraction form (Appendix 1, Supplementary material), inclusive of yes/no checkbox answers, single or multiple response answers, and short answer questions was developed. Based on a systematic review of characteristics, attributes, and outcomes of allied health transition programs [37] and scanning of job advertisements, 26 main attributes were identified and used as a framework for data extraction (Table 2). Similar attributes were grouped for ease of data extraction, for example, clinical/critical reasoning, problem- solving, and decision-making skills. Other data extracted included date advertised, organisation, contact person, location (to determine rurality using the Modified Monash Model), sector, workload/time fraction, qualifications, key selection criteria, and any other benefits offered, for example, financial supports, free carparking etcetera. To ensure accuracy and consistency, 10 advertisements were selected, and data extracted by two authors (RM and LH). Any differences were discussed and resolved. All data were then extracted by RM and checked for accuracy by LH.

**Table 2:** Employee attributes used for data extraction

Employee Attributes	
Academic results Accountability or responsibility Attitude Autonomy or work independently Client focus or client outcomes Clinical/critical reasoning, problem-solving, or decision-making skills Collaboration Communication or interpersonal skills Computer skills Cultural alignment or right fit Customer service or business skills Empathy Feedback - receiving or giving	Flexibility Initiative Interest in career, promotion, or progression Manual, technical or hands on clinical skills Passion Rapport, relationship building, or networking skills Resilience Self-confidence Self-motivation or emotional intelligence Teamwork or team player Time management, organisational skills, or ability to work under pressure Willingness to learn Work ethic

### Data analysis

Content analysis of the data were performed to systematically review and interpret the information within the job advertisements to gain an understanding [38] of the employability requirements and requested attributes of the early-career physiotherapist. This was completed on the Question Pro^®^ [36] platform by downloading the extracted data as Excel spreadsheets which included descriptive statistical analysis of the total number of times an item was selected, percentages, means, standard deviations, and graphing of the results for each question. Cross-tabulation on QuestionPro^®^ [36] was used to analyse the MM categories and attributes. Text mining and word embedding algorithm software were not used, as the researchers wanted to read each advert to gain an in-depth understanding of the multiple employability requirements and attributes requested from employers in different health sectors in various locations, the language used, and formatting of the advertisements, including position descriptions and links to additional information.

## Results

### Advertising period and locality

The SEEK.com.au database search from October 2020 to March 2021 yielded 578 job advertisements applicable to early-career physiotherapists. A summary of the main characteristics are represented in Tables 3 and 4. Almost half of the job advertisements were recorded in the first two months of data collection: October (*n* = 146; 25.3%) and November (*n* = 141; 24.4%), followed by March (*n* = 86; 14.9%), January (*n* = 76; 13.1%), December (*n* = 66; 11.4%), and February (*n* = 63; 10.9%). Most jobs were located within New South Wales (*n* = 187; 32.3%), Queensland (*n* = 153; 26.4%), and Victoria (*n* = 128; 22.1%), with the least number of advertised jobs in the Northern Territory (*n* = 4; 0.7%) and Australian Capital Territory (*n* = 9; 1.6%). Over half of the jobs (*n* = 379; 65.6%) were re-advertised during the six month data collection period ranging from 100% in MM 6 and MM 7 to 55.8% in MM 1. The locality of most jobs were in MM 1 metropolitan cities (*n* = 428; 74.0%), followed by MM 2 regional centres (*n* = 47; 8.1%), MM 3 large rural towns (*n* = 39; 6.7%), MM 4 medium rural towns (*n* = 41; 7.1%), MM 5 small rural towns (*n* = 19; 3.3%), with the two least represented categories being MM 6 remote communities (*n* = 3; 0.6%), and MM 7 very remote communities (*n* = 1; 0.2%).

**Table 3:** Summary of job advertisement according to location (*n* = 578)

State	Metro	Regional	Rural			Remote		State Total
	MM 1	MM 2	MM 3	MM 4	MM 5	MM 6	MM 7	
NSW *n* (%)	139 (74.3)	5 (2.7)	21 (11.2)	15 (8.1)	7 (3.7)	0	0	187
QLD *n* (%)	110 (71.9)	26 (16.9)	3 (2.0)	10 (6.5)	3 (2.0)	1 (0.7)	0	153
Vic *n* (%)	96 (75.0)	8 (6.3)	8 (6.3)	13 (10.1)	3 (2.3)	0	0	128
WA *n* (%)	49 (84.5)	0	3 (5.2)	1 (1.7)	5 (8.6)	0	0	58
SA *n* (%)	25 (86.2)	0	2 (6.9)	2 (6.9)	0	0	0	29
Tas *n* (%)	0	6 (60.0)	2 (20.0)	0	1 (10.0)	0	1 (10.0)	10
ACT *n* (%)	9 (100)	0	0	0	0	0	0	9
NT *n* (%)	0	2 (50.0)	0	0	0	2 (50.0)	0	4
Total *n* (%)	428	47	39	41	19	3	1	578

Note: the % of job advertisements reflect the % of the total for each state across MM categories; NSW New South Wales; QLD Queensland; Vic Victoria; WA Western Australia; SA South Australia; Tas Tasmania; ACT Australian Capital Territory; NT Northern Territory

**Table 4:** Summary of job advertisement characteristics

	Metro	Regional	Rural			Remote		Total
	MM 1	MM 2	MM 3	MM 4	MM 5	MM 6	MM 7	
Type of Advertisement								
Employer *n* (%)	372 (74.4)	43 (8.6)	33 (6.6)	34 (6.8)	16 (3.2)	2 (0.4)	0	500
Recruiter *n* (%)	56 (76.7)	4 (5.5)	4 (5.5)	6 (8.1)	1 (1.4)	1 (1.4)	1 (1.4)	73
Rural Health Platform^1^*n* (%)	0	0	2 (40.0)	1 (20.0)	2 (40.0)	0	0	5
Sector								
Private practice *n* (%)	286 (78.1)	22 (6.0)	20 (5.5)	25 (6.8)	10 (2.7)	2 (0.6)	1 (0.3)	366
Aged care *n* (%)	87 (69.6)	11 (8.8)	9 (7.2)	11 (8.8)	5 (4.0)	1 (0.8)	1 (0.8)	125
Community *n* (%)	65 (68.4)	14 (14.7)	3 (3.1)	9 (9.5)	2 (2.1)	1 (1.1)	1 (1.1)	95
Hospital *n* (%)	32 (71.1)	4 (8.9)	3 (6.7)	4 (8.9)	2 (4.4)	0	0	45
Disability *n* (%)	30 (81.1)	3 (8.1)	2 (5.4)	2 (5.4)	0	0	0	37
Other^2^*n* (%)	65 (67.0)	13 (13.4)	8 (8.2)	5 (5.2)	4 (4.1)	2 (2.1)	0	97
Target physiotherapist population								
Early-career *n* (%)	134 (70.9)	21 (11.1)	12 (6.3)	12 (6.3)	8 (4.2)	2 (1.2)	0	189
Experienced^3^*n* (%)	148 (76.7)	16 (8.3)	10 (5.2)	13 (6.7)	6 (3.1)	0	0	193
Both *n* (%)	146 (74.5)	10 (5.1)	17 (8.7)	16 (8.2)	5 (2.5)	1 (0.5)	1 (0.5)	196
Work fraction								
Full time *n* (%)	252 (73.7)	31 (9.1)	28 (8.2)	22 (6.4)	8 (2.3)	1 (0.3)	0	342
Part time *n* (%)	168 (76.0)	18 (8.1)	15 (6.8)	15 (6.8)	4 (1.8)	1 (0.5)	0	221
Locum *n* (%)	36 (58.1)	7 (11.3)	9 (14.5)	5 (8.1)	3 (4.8)	1 (1.6)	1 (1.6)	62
Casual *n* (%)	21 (72.4)	3 (10.3)	3 (10.3)	1 (3.5)	1 (3.5)	0	0	29
Not stated *n* (%)	75 (75.0)	6 (6.0)	2 (2.0)	10 (1.0)	6 (6.0)	1 (1.0)	0	100

Note: the % of job characteristics reflect the % of the total for each characteristic across MM categories; ^1^ Rural health workforce is a website/organisation that advertises rural jobs throughout Australia; ^2^ other includes not for profit, public & private organisations, general practice clinics and primary care centres; ^3^ job was targeted at experienced practitioners, however, new graduate/early-career would be considered for the role

### Position sectors, who advertised, and employment fractions

Most roles were within private practice (*n* = 366; 63.3%) or aged care sectors (*n* = 125; 21.7.%). However, some advertised roles could be categorised under multiple sectors. Community/domiciliary (*n* = 95; 16.4%), privately owned organisations (*n* = 78; 13.5%), and the disability sector (*n* = 37; 6.4%) were the next most populated work sectors, followed by public (*n* = 25; 4.3.%) and private hospitals (*n* = 20; 3.5%), and not for profit organisations (*n* = 16; 2.8%). The least represented sectors were primary care / general practice clinics (*n* = 2; 0.3%) and public organisations (*n* = 1; 0.8%). Most roles were within multi-disciplinary teams (*n* = 391; 67.6%), including largely exercise physiology (*n* = 116; 20.1%), occupational therapy (*n* = 107; 18.5%), massage therapy (*n* = 78; 13.5%), dietetics (*n* = 54; 9.3%), podiatry (*n* = 51; 8.8%), Pilates professionals (*n* = 31; 5.4%), and nursing (*n* = 30; 5.2%). Most advertisements came directly from the employer (*n* = 500; 86.5%), with 73 from a recruitment company (12.6%), and five from a rural health workforce organisation (0.9%).

Most positions were full-time (*n* = 342; 59.2%), followed by part-time (*n* = 221; 38.2%), locum or temporary (*n* = 62; 10.7%), and casual (*n* = 29; 5.0%). Workload requirements were not stated in 100 (17.2%) job advertisements. Some positions offered multiple work fractions. Early-career physiotherapists were targeted by employers in 189 (32.7%) advertisements, 193 (33.3%) jobs targeted experienced practitioners (however, new graduate / early-career would be considered for the role), and 196 (34.0%) jobs targeted both early-career and experienced physiotherapists for the role. Key selection criteria were included in 527 (91.2%) of the 578 job advertisements, ranging from 80.5% in MM 4 to 94.7% in MM 5 and 100% in MM 6. Additional required attributes were listed outside of the formal key selection criteria in 459 (79.4%) advertisements, ranging from 66.7% in MM 6 to 80.8% in MM 1.

### The attributes of early-career physiotherapists

Data about the 26 attributes of the early-career physiotherapist were extracted from the 578 job advertisements, and are represented in Table 5, with client focus most frequently requested (*n* = 375, 64.9%), followed by communication / interpersonal skills (*n* = 340, 58.8%), and teamwork or team player (*n* = 313, 54.1%). The least requested attributes were empathy (*n* = 24, 4.2%), resilience (*n* = 14, 2.4%) and good academic results (*n* = 2, 0.3%).

**Table 5:** Attributes listed in job advertisements

Attribute	Requested in Advertisement *n*(%)
Client focus / outcomes	375 (64.9)
Communication / interpersonal skills	340 (58.8)
Team or teamwork or team player	313 (54.1)
Wanting to learn or learning	280 (48.4)
Rapport /relationship building /networking skills or similar	251 (43.4)
Passion	211 (36.5)
Self-motivation / emotional intelligence	185 (32.0)
Autonomy /autonomous / work independently	171 (29.6)
Work ethic	170 (29.4)
Accountability / responsibility	165 (28.5)
Manual, technical or hands on clinical skills	165 (28.5)
Customer service / business skills	160 (27.7)
Cultural alignment / right fit	141 (24.4)
Time management / organisational skills / ability to work under pressure	139 (24.0)
Collaboration / collaborative - not teamwork	136 (23.5)
Clinical / critical reasoning / problem-solving /decision-making skills	107 (18.5)
An interest in career / promotion / progression	94 (16.3)
Initiative	89 (15.4)
Flexibility	88 (15.2)
Attitude	83 (14.3)
Computer skills	68 (11.8)
Feedback - receiving or giving	55 (9.5)
Self confidence	50 (8.7)
Empathy	24 (4.2)
Resilience	14 (2.4)
Academic results	2 (0.3)

The most frequently requested attributes according to MM category are summarised in Table 6. There were similarities between the most requested attributes from MM 1 (metropolitan areas) and MM 2 (regional centres), with the same five attributes frequently listed but in a different order, including client focus, communication and interpersonal skills, team player, willingness to learn and rapport, relationship building, and networking skills. Client focus was the highest ranking attribute in MM 1 (n-=274, 64.0%), MM 2 (*n* = 35, 74.5%), MM 3 (*n* = 26, 66.7%), MM 5 (*n* = 16, 84.2%), and MM 6 (*n* = 3, 100% ). While MM 3 (large rural towns) and MM 4 were similar to MM 1 and MM 2, these rural centres also requested passion (MM 3, *n* = 14, 35.9%) and work ethic (MM 4, *n* = 14, 34.1%). MM 5 (small rural towns) and MM 6 (remote communities) introduced a greater variety of desired attributes in the job advertisement that were equally ranked, for example, work ethic and self-motivation (MM 5, *n* = 7, 36.8%) and cultural alignment and an interest in career, promotion, or progression (MM 6, *n* = 1, 33.3%). There were no attributes listed in the job advertisement for MM 7 (very remote).

**Table 6:** Most desired attributes by employers based on MM category

Most desirable attributes (*n* / % by MM)^1,2^						
		1	2	3	4	5
MM Category (number of advertisements)	***MM 1 (428)***	Client focused (274, 64.0%)	Communication or IP skills (255, 59.6%)	Team player (238, 55.6%)	Willingness to learn (209, 48.8%)	Rapport, relationship building, or networking skills (180, 42.1%)
	***MM 2 (47)***	Client focused (35,74.5%)	Team player (28,59.6%)	Communication or IP skills (25, 53.2%)	Willingness to learn (24, 51.2%)	Rapport, relationship building, or networking skills (22, 46.8%)
	***MM 3 (39)***	Client focused Communication or IP skills (26, 66.7%)	Team player (22, 56.4%)	Willingness to learn (20, 51.3%)	Rapport, relationship building, or networking skills (16, 41.0%)	Passion (14, 35.9%)
	***MM 4 (41)***	Communication or IP skills (24, 58.5%)	Rapport, relationship building, or networking skills (23, 56.1%)	Client focused (21, 51.2%)	Team player (20, 48.8%)	Work ethic (14, 34.1%)
	***MM 5 (19)***	Client focused (16, 84.2%)	Rapport, relationship building, or networking skills (10, 52.6%)	Passion (9, 47.4%)	Communication or IP skills (8, 42.1%)	Work ethic Self-motivation Collaboration Willingness to learn (7, 36.8%)
	***MM 6 (3)***	Client focused Work ethic (3, 100% )	Communication or IP skills Passion Autonomy Accountability or responsibility Customer service Time management^*^ Clinical reasoning^**^ Initiative (2, 66.7% )	Cultural alignment An interest in career, promotion, or progression (1,33.3% )	No further attributes listed	No further attributes listed
	***MM 7 (1)***	No attributes listed	No attributes listed	No attributes listed	No attributes listed	No attributes listed

Note. ^1^% are a total of the MM category; ^2^% across an MM will not equal 100 as one advertisement may request multiple attributes; where equal ranking of an attribute, the number and percentage apply to each separately; IP interpersonal skills; * Time management / organisational skills / ability to work under pressure; ** Clinical / critical reasoning / problem-solving /decision-making skills

## Discussion

The aims of this study were to address the gaps in the current literature about characteristics of the jobs advertised for early-career physiotherapists in Australia; to determine which attributes are most desired when employing an early-career physiotherapist; and identify if there is a difference in the attributes required in the early- career physiotherapist based upon workplace location.

### The challenge of physiotherapy recruitment

Many jobs were re-advertised over the six-month timeframe of the study, indicating that recruitment in all regions of Australia is an ongoing workforce issue, with high early-career attrition [39] and turnover rates of physiotherapists being among the highest of all health professions within Australia [40]. The demand for physiotherapists far outweighs the supply of available professionals [5] therefore, most job advertisements target both early-career (with additional support) and experienced physiotherapists for full time or flexible roles in multi-disciplinary teams that were mostly in private practice or aged care sectors, within this competitive market. The greater representation of metropolitan jobs in the advertisements reviewed is not unexpected due to the population density of the large major cities within the states of New South Wales, Queensland, and Victoria. Low numbers of advertisements from remote areas may be due to outsourcing of physiotherapy services to practices or organisations located within larger rural and regional areas, may be a result of healthcare service provision inequities within rural and remote Australia [41], and therefore less job availability. In a study by Farquhar and colleagues [42], a public-private partnership was developed between a private practice and large rural health organisation to overcome longstanding recruitment difficulties leading to undersupply of rural physiotherapy services. It is unknown whether partnerships such as this may have reduced the numbers of remote positions advertised in the open market, but this could be a focus for further research.

### The importance of solid recruitment practices

The recruitment process is complex and dynamic [43] and the use of key selection criteria, qualifications, skills, attributes, and knowledge that have been defined by the employer as the most essential for fulfilling the job requirements [44], forms a large component of selecting the candidate that is the right fit for the organisation [45]. In our study, criteria were included in most advertisements, yet not always appropriate for the level of the target audience or had not been adjusted for dual levels of experience, for example, requesting dry needling skills from a new graduate. Such advanced skills are only possessed by experienced physiotherapists with formal post graduate qualifications in Australia. It was apparent that some advertisements had been developed from previously used organisational templates for other roles within the organisation and had not been proofread to ensure accuracy and coherency of information. The use of grammar tools, such as Grammarly, have been recommended to assist in word choice, avoid mistakes, and increase engagement of prospective incumbents [46]. 

Reading an advertisement and extracting the most relevant job-specific information is an important skill in applying for jobs [47]. Attributes required by employers were in the title, under key selection criteria (essential or desirable), listed as key responsibilities, anywhere within the summary, or in greater detail in position descriptions available from a hyperlink. The inexperienced physiotherapist would need to carefully scrutinise the advertisement to determine the true requirements of the role and determine if the values and mission of the organisation fit with their own attributes, and if not, self-select out before recruitment [48]. 

The digitisation of job advertisements has transformed the recruitment of potential employees by providing easily accessible job market information and application processes. Organisations have had to adjust to the use of communication technologies such as social media and job posting websites for online recruiting [49]. Online job advertisements provide a good source of information to identify and understand specific skill and attribute requirements of employees, however, do not necessarily provide an accurate account of the vacancy rates within a profession [50]. SEEK.com.au is a fee-for-service employment platform that allows employers to market their jobs online to gain a greater audience. In this study, it was evident that varying amounts of information were posted within the advertisement depending upon the package purchased by the employer. This may bias companies and small businesses that lack financial resources to purchase expansive online recruitment packages [5] or provide adequate information in their advertisements due to word limitations, often dictated by cost, to attract the best candidate.

### Attributes of early-career physiotherapists

This is the first study to use job advertisements to identify the most desirable attributes sought when employing an early-career physiotherapist and makes a major contribution to new knowledge. Exploration of attributes in physiotherapy are limited. Previous studies have focused on workplace readiness [18], the role of universities in preparing physiotherapists to meet the requirements of employers [19], attributes required for entry-level physiotherapists [19], exploration of single attributes [21–25], and differences between the attributes of early-career and experienced physiotherapists [21, 23, 26, 27], or postgraduate attributes [28]. By taking a broader approach, we have captured useful information on the most important attributes appearing in Australian job advertisements and the difference in attributes required in the early-career physiotherapist based upon workplace location.

The five most requested attributes of an early-career physiotherapist in the job advertisements reviewed were being client focused, having good communication and interpersonal skills, being a team player, having a willingness to learn, and being able to build rapport, relationships, and networking skills. In a large descriptive analysis of 142,000 online job advertisements for degree-qualified professionals, across a diversity of employment sectors, Rios and colleagues [5] identified the three most desirable attributes sought by employers in the United States: good communication (verbal and written), being collaborative, and having problem-solving skills. Collaboration and problem-solving, or clinical reasoning did not feature in the top five in our study, however, they did appear within our top 20 early-career physiotherapy attributes. Rios et al. [5]. , reinforced the need to translate their findings by focusing on 21st century learning skills to better prepare graduates to meet workplace expectations. They argued that the high ranking of communication skills, might indicate that employers perceive a skills gap amongst graduates in both written and oral communication which is why they rank so highly. They argue that student learning outcomes should align with employer identified attributes. We make a similar call and suggest that the findings of our study might be useful in curricula design and delivery. A major finding of Rios and colleagues [5] was that there were differences in the attributes required in different professional fields, with problem-solving and collaboration ranked much more highly in social science fields than business.

In a systematic review [51] of essential employability skills in medical services, the top five skills identified included, communication, computer skills, work psychology, teamwork, and interpersonal skills. Differences in the most highly ranked attributes in our study suggest that attributes sought by employers do differ between professional groups. However, this requires further exploration using different research methods. From our study, it is difficult to definitively state that the attributes listed in job advertisements are strongly aligned with the most important attributes required in practice. In-depth mixed methods studies, incorporating both survey and qualitative data collection, would enable a richer picture to be built about the alignment of what is advertised and what is needed in practice. Importantly, in-depth studies of this type would contribute to university course planning to ensure 21st century learning skills align with 21st century workplace skills [7].

Academic results are a focus of tertiary education, yet in our study, were not shown to be important to employers in the recruitment of new physiotherapists. Learning opportunities are often overshadowed by the emphasis placed on grades by the student, university [52], and clinical placement institutions. Ongoing development of personal and professional attributes from the university level and beyond is needed to prepare the healthcare workforce for the demands of the role, and to ensure long-term success [5]. This again, needs far greater exploration to ensure learning and teaching priorities align with industry need.

Empathy was not considered to be an essential attribute when advertising for an early- career physiotherapist, yet it is a vital component of person-centred care and understanding the needs of patients / clients. Rodriguez-Nogueira and colleagues [53] contend that universities should stress the importance of empathy in clinical practice and employers should provide education programs to educate physiotherapists in emotional management and empathetic strategies to avoid burnout and improve retention. In our study, resilience was not identified as an essential attribute to possess. In novice nurses, resilience has been identified as essential [54, 55] to job satisfaction and retention. Although we have highlighted attributes that were not strongly evident in job advertisements, it is unknown whether employers do not value these highly, or whether they make assumptions that these are fundamental qualities that all graduating physiotherapy students possess. Again, this is an area for further research.

### Differences in attributes in different workplace locations

Workplace locations for early-career physiotherapists are changing, with an expansion from public hospital-based settings to private practice environments [14], community settings, and primary care [15]. Job vacancies are advertised across a diversity of geographic settings from inner metropolitan to the most remote. Our study provides some insight into differences of advertised attributes contingent upon context.

The attributes of being client focused, having good communication and interpersonal skills, and teamwork were within our top five requested attributes for MM 1 to 4 categories, however, the smaller rural areas (MM 5) and remote areas (MM 6–7) of Australia do not highlight teamwork as an essential (top five) attribute to possess. It could be postulated that there are a greater number of sole practitioners in small organisations within these regions [56] making working in a physiotherapy team less likely, or that other attributes such as flexibility, autonomy [20], self-motivation, and collaboration with the patient and community, are considered more important when working in resource-limited environments. While attributes such as rapport and relationship building and having a good work ethic were considered more important with increasing rurality, this might align with studies that have shown the importance of developing trusting relationships and connections, both inside and outside the rural workplace [57], were vital for the healthcare professional’s integration into the community. There is a need for greater exploration of rural and remote differences because of the critical need for retention in these contexts.

Between August 2013 and July 2015, Onnis [7] took a similar approach to our study by collecting advertisements from five recruitment websites. Using content analysis, advertisements were systematically analysed to ascertain whether recruitment advertising aligned with the factors that influenced current health professionals to work in remote practice. While this work is useful in informing remote recruitment, the most important finding was that much work is needed to translate attraction into retention. While our study differed from that of Onnis [7], as our focus was on attributes, rather than the alignment of advertising with health professional need, Onnis’s work is useful in interpreting our findings. Onnis [7] highlighted the critical importance of ensuring that what is advertised reflects the reality of the role so that expectations of living and working in rural and remote settings are clear. The rural and remote context can be challenging with a lack of clinical and manager support, high workloads and long hours, limited resources, barriers to professional development, lack of professional and personal boundaries, and social isolation frequently documented [7, 16, 17]. Ensuring that recruitment materials reflect this reality is a key factor in retention [6], particularly for early-career physiotherapists who might have limited experience in rural and remote locations but have applied because they perceive jobs to be easier to obtain [17]. 

Specific attributes that are context-specific are important to capture, as identification of what is most valued might support the identification of priorities for mentoring, and support of newly qualified employees to further develop these attributes. Tailored professional development, mentoring, and support have been identified as central to the retention of health professionals [16, 17]. Further investigation of the link between the identification of attributes, use in recruitment, and development of attributes in the workplace, particularly in rural and remote locations would add to the workforce knowledge base.

More new graduate physiotherapists are entering private practice settings rather than the public hospital system [14], and employers are seeking passionate individuals. The medical profession has identified that society requires physicians that are passionate about their role and engaged in their work life [58]. In our study it was apparent in some job advertisements, that passion is a preferred attribute for early-career physiotherapists. The attributes of autonomy, time management, and organisational skills were more prevalent in private practices with greater rurality. It has been noted that rural practice offers greater autonomy but often comes hand in hand with greater responsibilities [16, 17, 59], hence requiring more highly developed time management and organisational attributes.

A greater proportion of the advertisements from this study were from metropolitan cities (MM 1), which may provide an uneven representation of the employment attributes from other MM categories.

Using an online portal such as SEEK.com.au may not capture all available advertisements. Other online sites such as LinkedIn, also provide a forum for advertising physiotherapy roles, therefore some jobs may not have been captured in this study. Rural and remote areas of the country find recruitment of physiotherapy staff challenging and may advertise via alternate methods after poor uptake using conventional methods, for example, word of mouth. These vacancies were not captured in this study.

In Australia, the State of New South Wales has a Physiotherapy New Graduate Allocation Program [60]. The program coordinates and allocates new graduate positions across diverse settings. It is not compulsory, but many hospital and rural secondment roles within the State are not advertised in the public space, and therefore were not captured in this study.

There are inherent limitations in drawing strong conclusions from studies using job advertisements. As noted, recruitment materials are only part of the picture in understanding attributes that are highly valued by employers in different settings. While our study does provide useful insights, an in-depth mixed methods study using surveys directly to employers and qualitative interviews would strengthen the conclusions that can be drawn. Research that explores the correlation between recruitment materials and the reality of roles and context from the perspective of employers and employees would add value.

## Conclusion

The findings of this study have several implications for the field of physiotherapy, particularly in the context of recruitment. Employers should critically assess the attributes they prioritise in their job advertisements and ensure that these align with the practical demands of the role. The challenges and unique requirements of different workplace locations should be considered, particularly in rural and remote areas, and recruitment strategies and job advertisements should be tailored accordingly. Developing comprehensive induction and mentoring programs, especially for early-career physiotherapists entering rural and remote settings, can aid in the retention and professional development of these professionals. Ultimately greater transparency and clarity in what these roles entail prior to appointment could enhance retention and would contribute to the improvement of healthcare delivery in underserved regions.

To facilitate a more comprehensive understanding of the dynamics between job advertisements, the actual workplace environment, and opportunities for this knowledge to inform educational design and delivery, future researchers should employ mixed methods, including surveys and qualitative interviews with both employers and employees. This would provide a more nuanced understanding of the discrepancies, if any, between the attributes sought in job advertisements and the actual requirements of the roles. Additionally, further investigation into the development of specific attributes, particularly in rural and remote settings, could aid in the creation of targeted education and support programs for physiotherapists practicing in these challenging environments. Overall, this study highlights the complex nature of physiotherapy recruitment in Australia and underscores the need for a more holistic approach to the identification and development of attributes that are crucial for the success and retention of early-career physiotherapists. By addressing the gaps identified in this research, both educational institutions and employers can play a pivotal role in enhancing the quality of care provided by physiotherapists, and in turn, improve the overall healthcare landscape in Australia.

## Electronic supplementary material

Below is the link to the electronic supplementary material.

Supplementary Material 1

## Data Availability

The datasets used and/or analysed during the current study are available from the corresponding author on reasonable request.
