# Supplementary material for: Employers most desirable attributes in early-career physiotherapists: a content analysis of job advertisements
Source: BMC Health Serv Res. 2024 Sep 6;24:1038. doi: 10.1186/s12913-024-11470-6 (PMC11380398; doi:10.1186/s12913-024-11470-6)
Supplement: Supplementary file 1 — Supplementary Material 1 [file 12913_2024_11470_MOESM1_ESM.docx]

**Appendix 1** QuestionPro data extraction questions

| Job Ad Code |
| --- |
| Which month was the job advertised? |
| Which State or Territory is the job advertised? |
| Where is the main location of the job? |
| What is the postcode of the main location? |
| What is the location’s Modified Monash Rurality score? |
| What is the name of the advertising organisation? |
| Is the advertising organisation a recruitment agency? |
| Is there a contact person? |
| If yes, what is the contact person's name? |
| Is there a contact email? |
| If yes, what is the email address? |
| Is there a contact phone number? |
| If yes, what is the contact phone number? |
| What is the organisation's web address |
| Was the web address provided on the advertisement? |
| What sector is the job within? |
| Is the organisation multi-disciplinary (from the information provided?) |
| Which professions are employed by this organisation? |
| What is the workload? |
| What is the case load? |
| Who is the main target physiotherapist for this advertisement? New graduate (NG) / early career (EC); Experienced; Both equally encouraged to apply. |
| Will they accept new graduates or are new graduates encouraged to apply, but are not considered the main target? Yes/No |
| Apart from a recognised Austrailan physiotherapy degree, what other qualifications are requested? National police check; CPR/first aid; Post graduate qualifications or working towards; Flu vaccine; Covid vaccine; Working with Children Check; None stated; Other |
| Will the organisation support oversea physiotherapists with sponsorship? Yes/No/Not stated |
| Please select the listed registration requirements in the advertisement – Full Australian Health Practitioner Regulation Agency;  Pending Australian Health Practitioner Regulation Agency; Eligibility to work in Australian / working rights; Unrestricted working rights; None stated: Other |
| Does the applicant require professional indemnity insurance? Yes/No/Not stated |
| What are the key selection / essential criteria (must be listed as such, not info in the blurb)? |
| Are further criteria throughout the advertisement (in the blurb) but not under the heading of key selection criteria? Yes/No |
| Is the applicant required to have a driver’s license? Yes/No/Not stated |
| Is the applicant required to have a car? Yes/No/Not stated/Other |
| Are required or desired physiotherapy skills listed? Yes/No |
| Attributes: Rapport / relationship building /networking skills or similar  (is it mentioned in the advertisement?) Yes/No. Please add further specific information as relevant |
| Attributes: Communication (is it mentioned in the advertisement?) Yes/No.  Please add further specific information as relevant . |
| Attributes: Communication- what words were used in relation to this attribute? Excellent / Exceptional / Interpersonal / People skills / Verbal / Written / Strong / Proficient / With other health professionals / With patients/clients / To form relationships / Open /Exceptional / Advanced / Listening / Other |
| Attributes: Autonomy/autonomous / work independently (is it mentioned in the advertisement?) Yes/No  Please add further specific information as relevant. |
| Attributes: Collaboration/collaborative - not teamwork (is it mentioned in the advertisement?) Yes/No  Please add further specific information as relevant. |
| Attributes: Team or teamwork or team player (is it mentioned in the advertisement?) Yes/No  Please add further specific information as relevant. |
| Attributes: Client focus / outcomes (is it mentioned in the advertisement?) Yes/No  Please add further specific information as relevant. |
| Attributes: computer skills (is it mentioned in the advertisement?) Yes/No  Please add further specific information as relevant. |
| Attributes: Initiative - (is it mentioned in the advertisement?) Yes/No  Please add further specific information as relevant. |
| Attributes: Self-motivation / emotional intelligence   - (is it mentioned in the advertisement?)  Yes/No. Please add further specific information as relevant. |
| Attributes: Self-confidence - (is it mentioned in the advertisement?) Yes/No  Please add further specific information as relevant. |
| Attributes: Attitude -  (is it mentioned in the advertisement?)  Yes/No  Please add further specific information as relevant. |
| Attributes: Accountability / responsibility -  (is it mentioned in the advertisement?)   Yes/No  Please add further specific information as relevant. |
| Attributes: Feedback - receiving or giving (is it mentioned in the advertisement?) Yes/No  Please add further specific information as relevant. |
| Attributes: Wanting to Learn or learning (is it mentioned in the advertisement?) Yes/No  Please add further specific information as relevant. |
| Attributes:  An interest in career / promotion / progression (is it mentioned in the advertisement?) Yes/No  Please add further specific information as relevant. |
| Attributes: Resilience (is it mentioned in the advertisement?) Yes/No  Please add further specific information as relevant. |
| Attributes: Empathy (is it mentioned in the advertisement?) Yes/No  Please add further specific information as relevant. |
| Attributes: Passion (is it mentioned in the advertisement?) Yes/No  Please add further specific information as relevant. |
| Attributes: Work ethic - (is it mentioned in the advertisement?) Yes/No  Please add further specific information as relevant. |
| Attributes: Flexibility - (is it mentioned in the advertisement?) Yes/No  Please add further specific information as relevant. |
| Attributes: Cultural alignment / right fit - (is it mentioned in the advertisement?) Yes/No  Please add further specific information as relevant. |
| Attributes: Time management / organisational skills / ability to work under pressure- (is it mentioned in the advertisement?) Yes/No  Please add further specific information as relevant. |
| Attributes: Clinical / critical reasoning / problem solving /decision making skills- (is it mentioned in the advertisement?) Yes/No  Please add further specific information as relevant. |
| Attributes: Academic results- (is it mentioned in the advertisement?) Yes/No  Please add further specific information as relevant. |
| Attributes: Customer service / business skills- (is it mentioned in the advertisement?) Yes/No  Please add further specific information as relevant. |
| Attributes: Other attributes listed anywhere within the advertisement. Please select as many as relevant: Fun; Sense of humour; Energetic; Vibrant; Friendly; Confident; Outgoing; Dynamic; Caring; Dedicated; Positive; Likeminded; Professional; Ethical; Pro-active; Driven; Enthusiastic; Awesome; Other |

*Note.* Only questions relevant to this publication are included.
